# Development of Alginate Microspheres Containing Chuanxiong for Oral Administration to Adult Zebrafish

**DOI:** 10.1155/2016/4013071

**Published:** 2016-06-15

**Authors:** Li-Jen Lin, Chung-Jen Chiang, Yun-Peng Chao, Shulhn-Der Wang, Yu-Ting Chiou, Han-Yu Wang, Shung-Te Kao

**Affiliations:** ^1^School of Chinese Medicine, College of Chinese Medicine, China Medical University, Taichung 40402, Taiwan; ^2^Department of Medical Laboratory Science and Biotechnology, China Medical University, Taichung 40402, Taiwan; ^3^Department of Chemical Engineering, Feng Chia University, Taichung 40724, Taiwan; ^4^Department of Health and Nutrition Biotechnology, Asia University, Taichung 41354, Taiwan; ^5^School of Post-Baccalaureate Chinese Medicine, College of Chinese Medicine, China Medical University, Taichung 40402, Taiwan; ^6^Department of Chinese Medicine, China Medical University Hospital, Taichung 40402, Taiwan

## Abstract

Oral administration of Traditional Chinese Medicine (TCM) by patients is the common way to treat health problems. Zebrafish emerges as an excellent animal model for the pharmacology investigation. However, the oral delivery system of TCM in zebrafish has not been established so far. This issue was addressed by development of alginate microparticles for oral delivery of chuanxiong, a TCM that displays antifibrotic and antiproliferative effects on hepatocytes. The delivery microparticles were prepared from gelification of alginate containing various levels of chuanxiong. The chuanxiong-encapsulated alginate microparticles were characterized for their solubility, structure, encapsulation efficiency, the cargo release profile, and digestion in gastrointestinal tract of zebrafish. Encapsulation of chuanxiong resulted in more compact structure and the smaller size of microparticles. The release rate of chuanxiong increased for alginate microparticles carrying more chuanxiong in simulated intestinal fluid. This remarkable feature ensures the controlled release of encapsulated cargos in the gastrointestinal tract of zebrafish. Moreover, chuanxiong-loaded alginate microparticles were moved to the end of gastrointestinal tract after oral administration for 6 hr and excreted from the body after 16 hr. Therefore, our developed method for oral administration of TCM in zebrafish is useful for easy and rapid evaluation of the drug effect on disease.

## 1. Introduction

Chuanxiong is a Traditional Chinese Medicine (TCM) herb and also a common dietary in Asia. It contains more than 200 isolated compounds, and tetramethylpyrazine is identified as one of the most active ingredients [1, 2]. The high medicinal value of chuanxiong can be acknowledged by its ability to promote blood circulation and alleviate pain [3]. This herb has been widely used in treatment of cardiovascular disease, atherosclerosis [4], and thrombus formation [5]. In particular, chuanxiong exhibits the antifibrotic and antiproliferative effects on hepatocytes as reported by* in vitro* and* in vivo* studies [6, 7].

Chronic hepatitis B virus (HBV) and C virus (HCV) infections are usually associated with the progression of hepatic fibrosis [8]. HBV and HCV are involved in the formation of intrahepatic cholangiocarcinoma (ICC), and coinfection of both viruses increases the severity of hepatitis and hepatic fibrosis [8, 9]. ICC is the second most common hepatic malignancy with poor prognosis and a low resectability rate [8]. To study this hepatic disease, the transgenic model of ICC has been developed by coexpression of HBV X and HCV core protein in zebrafish [8]. This model displays similar histopathology and molecular features including neoplasm networks and potential biomarkers to human ICC. Apparently, it facilitates the investigation on the effect of therapeutic drugs on HBV- and HCV-induced fibrosis and bile duct neoplasms.

Zebrafish is recognized as an excellent animal model for gene studies and toxicity analyses of vertebrates. In particular, the human disease states are usually mirrored by the mutant phenotypes of zebrafish. Therefore, the development of transgenic zebrafish models for human diseases offers a promising means to investigate the corresponding pathophysiology and screen potential therapeutic drugs [10]. To analyze the drug effects, adult zebrafish are conventionally given medication by direct bathing the drugs in a chemical reagent or by peritoneal injection of the drugs. Note that TCM is administrated orally by human patients. It is obvious that a new delivery system of TCM is required for studies of their effects in the zebrafish models. This issue was addressed by using alginate for oral delivery of chuanxiong. Alginate is a polymer with high biological compatibility and has been used for encapsulation of drugs, proteins, and cells [11–13]. In this study, the alginate-based delivery system was developed for oral administration of chuanxiong in zebrafish. Our results show that this delivery system has a potential for rapid selection of TCM for effective treatment of diseases.

## 2. Materials and Methods

### 2.1. Zebrafish and Reagents

The experiments using the zebrafish AB strain were carried out in agreement with the principles outlined by the Committee of China Medical University (Number 103-232-C). The zebrafish strain was maintained in light cycle of 14 hr light/10 hr dark at 25°C. Chuanxiong (Kaiser Pharmaceutical Co., Ltd., Taiwan) was dissolved in distilled water for encapsulation by alginate microparticles and stored at −20°C until use. Alginate and TetraBits® Complete (fish food) were purchased from Chuang Song Zong Pharmaceutical Co., Ltd. (Kaohsiung, Taiwan) and Tetra GmbH (Germany), respectively.

### 2.2. Encapsulation of Chuanxiong by Alginate Microparticles

The dose of chuanxiong is based on the doctors' prescriptions for liver cancer treatment in Taiwan. A daily dose for an adult is 1x chuanxiong (14.29 mg/kg). Alginate microparticles were prepared by dropwise addition of the alginate solution (15 mL) containing 1% alginate and 1.5 g of fish food into 100 mL calcium chloride (1.5%) solution with magnetic stirring. Microparticles were formed and then left in the solution for 10 min at room temperature. Similarly, the chuanxiong-loaded alginate microparticles were prepared using the alginate solution containing 0.5x, 1x, or 1.5x chuanxiong. The sizes of alginate microparticles free of or encapsulated with chuanxiong were determined by optical microscope system (Carl Zeiss Axioskop 2, Germany).

### 2.3. Characterization of Alginate Microparticles

The swelling behavior of free or chuanxiong-loaded alginate microparticles was investigated by soaking dried ones into 200 *μ*L water or simulated intestinal fluid (pH 6.8) in the 96-well plate for 24 hr. The composition of simulated intestinal fluid contained 6.8 g of monobasic potassium phosphate and 0.2 N NaOH (190 mL) per L. Microparticles were sampled and their sizes were then determined at time intervals. The swelling rate of microparticles was calculated as follows:

(1) $$\begin{array}{c} \text{Swelling rate}=\frac{\left(\text{diameter of swollen microparticle}-\text{diameter of dried microparticle}\right)}{\text{diameter of dried microparticle}}. \end{array}$$

In addition, the morphology of alginate microparticle was examined by scanning electron microscopy (SEM). Followed by dehydration using Critical Point Dryer (CPD), one microparticle was attached to aluminum stubs and examined in a field emission scanning electron microscope (JEOL JSM-7401F). Moreover, one free or loaded alginate microparticle was clamped and its size was measured. The shape change of microparticle was calculated as follows:

(2) $$\begin{array}{c} \text{Shape change rate}=\frac{\left(\text{diameter of clamped microparticle}-\text{diameter of microparticle}\right)}{\text{diameter of microparticle}}\times 100\%. \end{array}$$

### 2.4. *In Vitro* Release Study of Alginate Microparticles

The* in vitro* release study was carried out with the chuanxiong-loaded alginate microparticles in water and simulated intestinal fluid. Microparticles (0.2 g) were placed in a 15 mL tube containing 2 mL of water or simulated intestinal fluid and incubated at room temperature with shaking at 100 rpm. The solution (0.5 mL) was sampled for determination of released chuanxiong by measuring ferulic acid using HPLC at time intervals. HPLC was equipped with Waters 515 HPLC Pump, Waters 2996 Photodiode Array Detector (PDA), and Waters SunFire*™* C18 5 *μ*m 4.6 × 250 mm column. The mobile phase consisted of 2% acetic acid in water/acetonitrile and the flow rate was set at 0.6 mL/min. A gradient elution was performed with 0–100% acetonitrile in 0~50 min and 100–0% acetonitrile in 55~60 min. The eluate was detected at 320 nm. The encapsulation and release efficiency of chuanxiong by alginate microparticles were calculated as follows:

(3) Encapsulation  efficiency=loaded  chuanxiongoriginal  chuanxiong×100%,Release  efficiency=released  chuanxiongloaded  chuanxiong×100%.

### 2.5. Oral Administration of Alginate Microparticles

Adult zebrafish were fasted overnight and randomly placed into tanks (one fish per tank) before oral administration of alginate microparticles which contained red dye (Widetex Co., Taiwan). Each fish was sacrificed at 0, 1, 3, 6, 9, and 16 h (*n* = 6 at each point) after oral administration. The gastrointestinal tract of fish was observed by optical microscope system (Carl Zeiss Axioskop 2, Germany) and fluorescence microscope (OLYMPUS, BX50, Japan) to assess consumption of microparticles.

### 2.6. Statistics

Data were reported as mean ± standard deviation (SD). The statistical analysis was analyzed by Student's *t*-test, and *P* value <0.05 indicated the statistical significance.

## 3. Results

### 3.1. Characteristic of Chuanxiong-Encapsulated Alginate Microparticles

**Figure 1:**
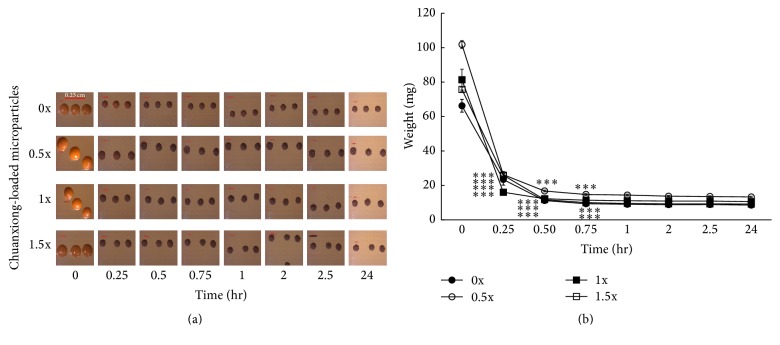
The effect of drying on the size and weight of chuanxiong-loaded alginate microparticles. (a) The size of microparticles free of (0x) or encapsulated with chuanxiong. The time before drying was denoted by “0.” (b) The weight of microparticles free of or encapsulated with chuanxiong. Values represent the mean ± SD of 10 microparticles per group. ^*∗∗∗*^ *P* < 0.001 (between after time and before time in the same group).

Alginate microparticles free of or encapsulated with various chuanxiong were prepared for studies. To facilitate the feeding of zebrafish, these microparticles were reshaped by drying in an incubator at 37°C. The sizes of microparticles were reduced by half after drying for 0.25 h and gradually shank from 0.25 h to 0.5 h (Figure 1(a)). After that, the size and the weight of microparticles remained almost unchanged. Dried microparticles exhibited the biggest size and the heaviest weight among all (Figure 1(b)).

**Figure 2:**
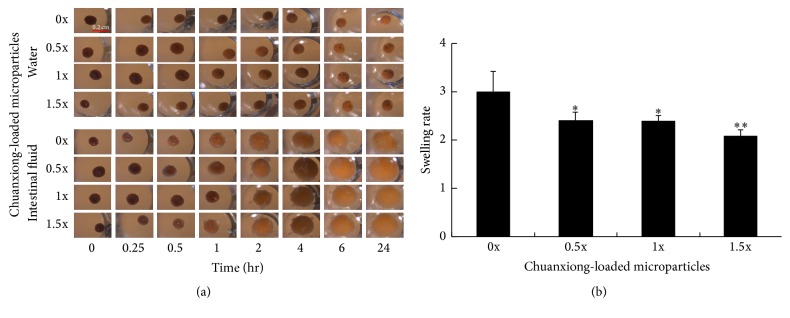
The swelling behavior of chuanxiong-loaded alginate microparticles. (a) The size of alginate microparticles free of or encapsulated with chuanxiong in water and simulated intestinal fluid. (b) The swelling rate of microparticles were compared with the same microparticle size between 24 hours and 0 mins in simulated intestinal fluid. Values represent the mean ± SD. ^*∗*^ *P* < 0.05; ^*∗∗*^ *P* < 0.01 (versus 0x group).

Dried microparticles were soaked into water and stimulated intestinal fluid. No swelling for these microparticles was observed in water (Figure 2(a)). In contrast, microparticles swelled in the simulated intestinal fluid and their sizes increased along the time course. The size of microparticles free of chuanxiong was increased by 3-fold while the size of chuanxiong-encapsulated microparticles was approximately doubled (Figure 2(b)).

### 3.2. Morphology of Chuanxiong-Encapsulated Alginate Microparticles

**Figure 3:**
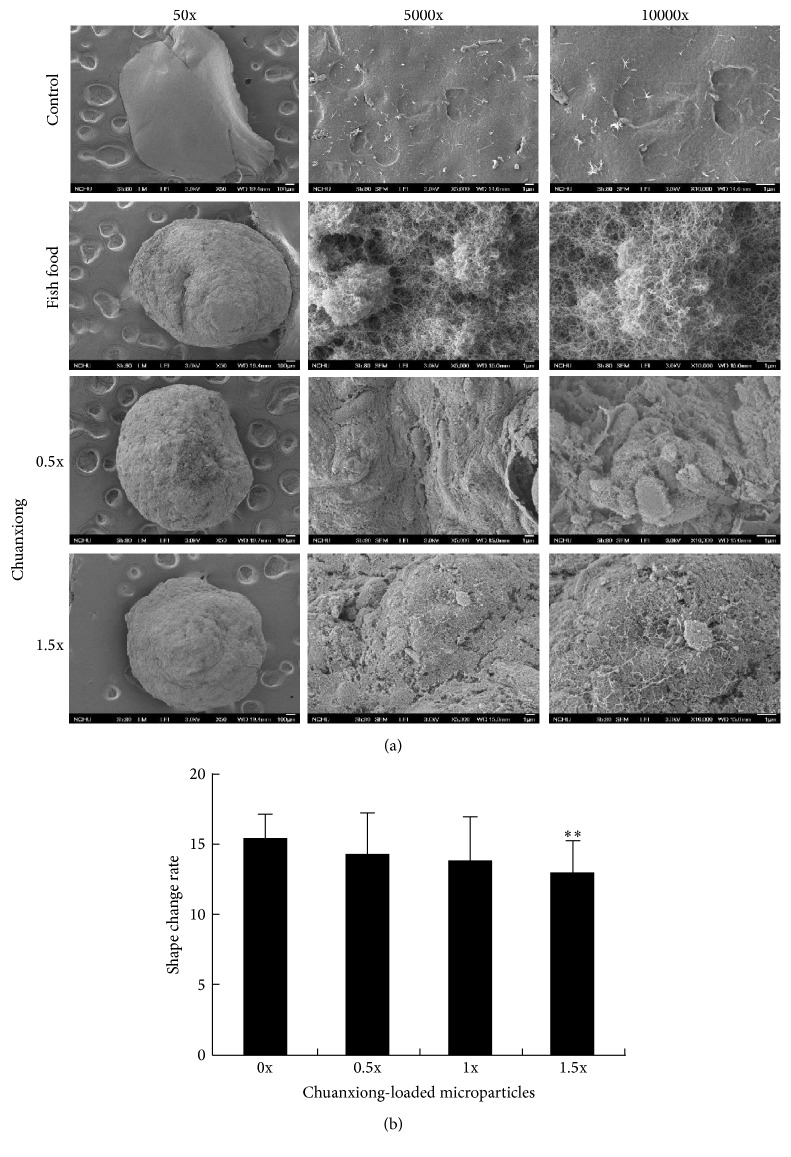
The effect of chuanxiong on the structure of alginate microparticles. (a) The SEM analysis with amplification at 50x, 5000x, and 10000x. (b) The shape change rate of microparticles free of or encapsulated with chuanxiong. Values represent the mean ± SD. ^*∗∗*^ *P* < 0.01 (versus 0x group).

The effect of encapsulated chuanxiong on microparticles structure was investigated by SEM. In comparison with pure alginate microparticles, microparticles loaded with fish food assumed a loose structure as revealed by the SEM analysis (Figure 3(a)). The additional encapsulation of chuanxiong rendered the structure of microparticle more compact. In addition, the shape change rate of alginate microparticles decreased with the increasing level of encapsulated chuanxiong (Figure 3(b)). Based on the definition of the shape change rate, the result indicates that the structure of alginate microparticles after encapsulation with chuanxiong becomes more stretchable.

### 3.3. *In Vitro* Release Profiles

**Figure 4:**
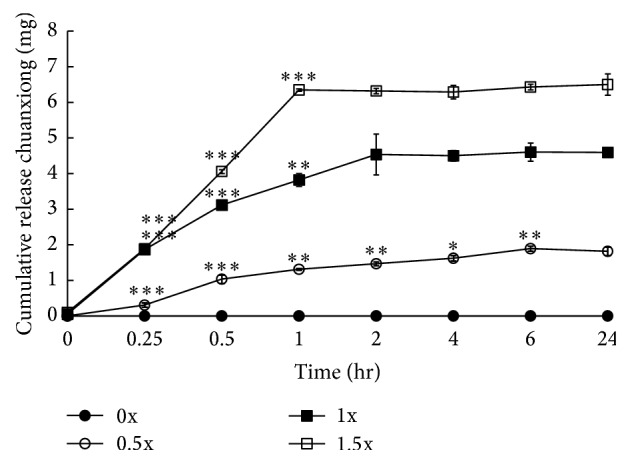
The release efficiency of chuanxiong-loaded alginate microparticles. Values represent the mean ± SD. ^*∗*^ *P* < 0.05; ^*∗∗*^ *P* < 0.01; and ^*∗∗∗*^ *P* < 0.001 (between after time and before time in the same group).

The release profile of chuanxiong-encapsulated alginate microparticles was studied* in vitro*. As shown in Figure 4, chuanxiong was released from alginate microparticles along the time course. Microparticles with 1.5x chuanxiong exhibited the most rapid release of the herb cargo compared to others. There was 11.5% of the loaded chuanxiong (ca. 29.22 *μ*g/macroparticle) released from 1.5x chuanxiong-encapsulated microparticles at 24 h (Table 1). Interestingly, microparticles with 0.5x chuanxiong exhibited a prolonged release profile. Moreover, the encapsulation efficiency of chuanxiong-loaded microparticles reached 76.72%–74.29%. As estimated, oral administration of 2 microparticles containing 0.5x, 1x, or 1.5x chuanxiong per day for one fish could reach the maximum dosage of chuanxiong (Table 1).

### 3.4. Oral Administration of Chuanxiong-Encapsulated Alginate Microparticles

**Figure 5:**
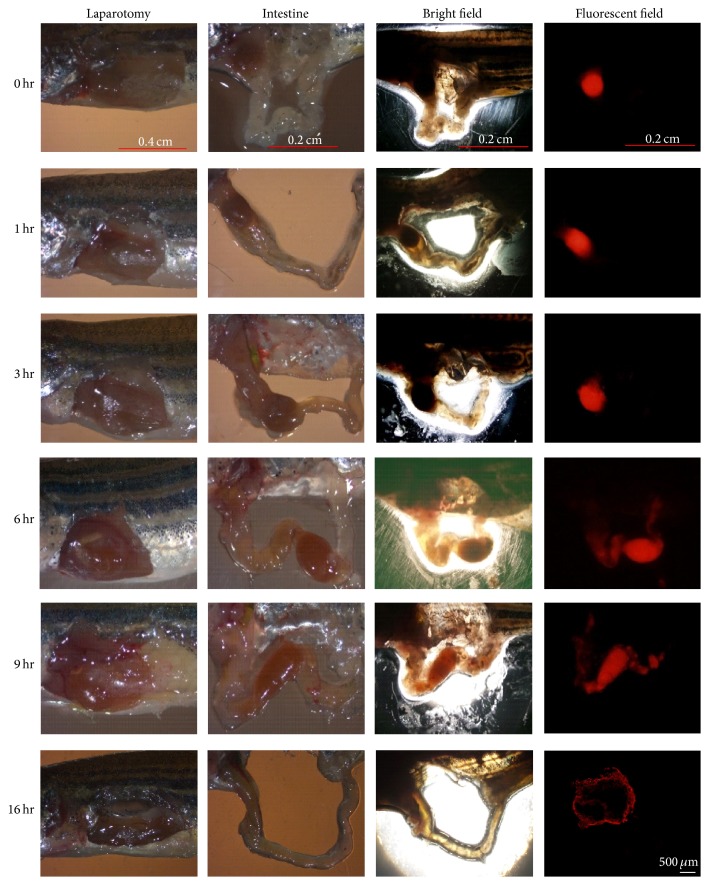
The analysis of chuanxiong-loaded alginate microparticles in zebrafish intestine. The microparticles were excreted after 16 h as shown in the fluorescent field.

Finally, oral administration of alginate microparticles was carried out in the AB line zebrafish twice a day (in the morning and afternoon). It was found that the average time for uptake of one microparticle with no, 0.5x, 1x, and 1.5x chuanxiong by a single fish was 47, 23, 31, and 34 seconds (*n* = 40), respectively. Moreover, the gastrointestinal tract of zebrafish was observed after administration of the 1.5x chuanxiong-loaded microparticles (Figure 5). Microparticles were digested in chylomicrons after 9 h and excreted from the body after 16 h.

## 4. Discussion

Carriers such as micelles, dendrimers, and microspheres are commonly employed for the oral delivery of drugs, and the receptor-, paracellular-, carrier-, M cell-, or/and transcellular-mediated adsorption of the loaded drugs proceed in intestinal epithelium [14]. The carrier materials usually involve chitosan, alginate, PLA, and PGA which are biodegradable as well as biocompatible and approved for human use by FDA [14, 15]. Moreover, these polymeric materials have flexibility in size, surface charge, and hydrophobicity and are endowed with the property for controlled release of drug [15]. In this study, the oral administration system of TCM in zebrafish was developed based on alginate. This was illustrated by encapsulation of chuanxiong with alginate. The resulting alginate microparticles were dried and utilized as fish feed. Dried alginate microparticles were insoluble in water and became swollen in simulated intestinal fluid (Figure 2). This remarkable feature ensures the controlled release of encapsulated drugs in the gastrointestinal tract of zebrafish. Loaded with more chuanxiong, alginate microparticles after drying had a smaller swollen size in stimulated intestinal fluid. It is likely that interaction of chuanxiong compositions with alginate results in more compact structure of microparticles. This view is supported by the SEM analysis, revealing that encapsulation of chuanxiong leads to more compact structure and the smaller size of microparticles (Figure 3).

The release of chuanxiong from alginate microparticles is unaffected by their compact structure. As shown in Figure 4, the release rate of chuanxiong increased for alginate microparticles carrying more chuanxiong and the released chuanxiong from microparticles with 0.5x, 1x, and 1.5x chuanxiong almost reached the plateau. It was estimated that 1.5 and 2 particles per day for one zebrafish achieved the minimum and maximum dosage for one patient according to the release profiles of alginate microparticles loaded with 3 levels of chuanxiong. Moreover, alginate microparticles were moved to the end of gastrointestinal tract after oral administration for 6 hr, digested in chylomicrons after 9 hr, and excreted from the body after 16 hr. Therefore, the encapsulated chuanxiong released from the ingested alginate microparticles could reach the effective dosage before being excreted. This model offers an advantage of the study on the disease treatment by TCM in zebrafish.

Cancer has become the most life-threatening illness worldwide. There are around 14.1 million diagnosed cases of cancer and 8.2 million people who die of cancers per year [16]. Apparently, it is important to have an efficient method for evaluation of the drug efficacy in cancer therapy. Most clinical trials have shown the usefulness of TCM for disease/cancer treatment [17–19]. In addition, over 70% of zebrafish genes share similarity with human genes [20] and zebrafish cancers are also similar to human cancers in the histological and genetic aspects [21]. We developed a new method to prepare alginate microparticles for oral delivery of chuanxiong (a TCM) which were stable and attractive to zebrafish to eat easily. The cumulative release chuanxiong of two chuanxiong-encapsulated alginate microparticles could reach clinical daily dosage before 6 hr in simulated intestinal fluid. It not only solves the oral administration zebrafish with TCM but also provides new field to explore therapy mechanism of TCM by disease model of transgenic zebrafish. Therefore, this technique may contribute to development of new drugs that are more suited for modern people.

## Figures and Tables

**Table 1:** Characteristics of chuanxiong-loaded alginate microparticles. Values represent the mean ± SD. ^*∗*^ *P* < 0.05; ^*∗∗*^ *P* < 0.01; and ^*∗∗∗*^ *P* < 0.001 (versus 0.5x group).

Chuanxiong-loaded microparticle	Encapsulation efficiency%	Release efficiency%	Drug loading	Daily dosage	
			Chuanxiong (*μ*g/microparticle)	Minima (microparticles/day)	Maxima (microparticles/day)
0.5x	76.72 ± 1.19	9.88 ± 0.54	8.70 ± 0.47	1.57 ± 0.08	2.35 ± 0.12
1x	73.35^*∗∗*^ ± 0.15	12.74^*∗∗∗*^ ± 0.23	23.09^*∗∗∗*^ ± 0.42	1.18 ± 0.02	1.77 ± 0.03
1.5x	74.29^*∗*^ ± 0.34	11.50^*∗*^ ± 0.44	29.22^*∗∗∗*^ ± 1.11	1.40 ± 0.05	2.10 ± 0.08
